# Red Propolis and Its Dyslipidemic Regulator Formononetin: Evaluation of Antioxidant Activity and Gastroprotective Effects in Rat Model of Gastric Ulcer

**DOI:** 10.3390/nu12102951

**Published:** 2020-09-26

**Authors:** Marcio A. A. de Mendonça, Ana R. S. Ribeiro, Adriana K. de Lima, Gislaine B. Bezerra, Malone S. Pinheiro, Ricardo L. C. de Albuquerque-Júnior, Margarete Z. Gomes, Francine F. Padilha, Sara M. Thomazzi, Ettore Novellino, Antonello Santini, Patricia Severino, Eliana B. Souto, Juliana C. Cardoso

**Affiliations:** 1 University of Tiradentes, Av. Murilo Dantas, 300, Aracaju CEP 49032-490, Sergipe, Brazil; andrade.mendonca@oi.com.br (M.A.A.d.M.); adrianaklima@live.com (A.K.d.L.); gislaine.proativa@yahoo.com.br (G.B.B.); malonepinheiro@hotmail.com (M.S.P.); ricardo.patologia@uol.com.br (R.L.C.d.A.-J.); guetezanardo@yahoo.com.br (M.Z.G.); fpadilha@yahoo.com (F.F.P.); pattypharma@gmail.com (P.S.); 2 Departament of Physiology, Federal University of Sergipe, Av. Marechal Rondon, Cidade Universitária, São Cristóvão CEP 49100-000, Sergipe, Brazil; ana_farmaunit@hotmail.com (A.R.S.R.); sthomazzi@gmail.com (S.M.T.); 3 Institute of Technology and Research (ITP), Av. Murilo Dantas, 300, Aracaju CEP 49032-490, Sergipe, Brazil; 4 Department of Pharmacy, University of Napoli Federico II, Via D. Montesano 49, 80131 Napoli, Italy; ettore.novellino@unina.it; 5 Tiradentes Institute, 150 Mt Vernon St, Dorchester, MA 02125, USA; 6 Department of Pharmaceutical Technology, Faculty of Pharmacy, University of Coimbra, Pólo das Ciências da Saúde, Azinhaga de Santa Comba, 3000-548 Coimbra, Portugal; 7 CEB—Centre of Biological Engineering, University of Minho, Campus de Gualtar, 4710-057 Braga, Portugal

**Keywords:** propolis, formononetin, dyslipidemia, gastric ulcer, rats

## Abstract

Propolis has various pharmacological properties of clinical interest, and is also considered a functional food. In particular, hydroalcoholic extracts of red propolis (HERP), together with its isoflavonoid formononetin, have recognized antioxidant and anti-inflammatory properties, with known added value against dyslipidemia. In this study, we report the gastroprotective effects of HERP (50–500 mg/kg, p.o.) and formononetin (10 mg/kg, p.o.) in ethanol and non-steroidal anti-inflammatory drug-induced models of rat ulcer. The volume, pH, and total acidity were the evaluated gastric secretion parameters using the pylorus ligature model, together with the assessment of gastric mucus contents. The anti-*Helicobacter pylori* activities of HERP were evaluated using the agar-well diffusion method. In our experiments, HERP (250 and 500 mg/kg) and formononetin (10 mg/kg) reduced (*p* < 0.001) total lesion areas in the ethanol-induced rat ulcer model, and reduced (*p* < 0.05) ulcer indices in the indomethacin-induced rat ulcer model. Administration of HERP and formononetin to pylorus ligature models significantly decreased (*p* < 0.01) gastric secretion volumes and increased (*p* < 0.05) mucus production. We have also shown the antioxidant and anti-*Helicobacter pylori* activities of HERP. The obtained results indicate that HERP and formononetin are gastroprotective in acute ulcer models, suggesting a prominent role of formononetin in the effects of HERP.

## 1. Introduction

Dyslipidaemia is a condition defined either by the high levels of total or low-density lipoprotein (LDL) cholesterol, and/or by the low levels of high-density lipoprotein (HDL). Such lipid metabolic dysfunction, resulting from, e.g., lipid peroxidation or oxidative stress, plays an important role in the pathogenesis of atherosclerosis and other chronic diseases [1]. Changes in lifestyle (e.g., adoption of a heart-healthy diet and exercise) and the use of drugs (e.g., fibrates to reduce triacylglycerols, statins to reduce bad cholesterol (LDL), and niacin to increase the overall (HDL) cholesterol) are commonly implemented to ameliorate these metabolic diseases. Atherogenesis results from the imbalance between the antioxidant capacity and the activity of oxidative species (reactive oxygen, nitrogen, and halogen). The oxidizing cellular protein, lipid, and DNA directly injure cells or these can even die from disturbances in signaling pathways. Reactive oxygen species (ROS) participate as signaling molecules in fundamental cellular functions in ambient conditions. Oxidative stress, and activation of ROS, is triggered when a cell is depleted in small molecule antioxidants or when antioxidase systems are overloaded or not operating at all.

The interest in natural products as sources of new drugs and therapeutic strategies for chronic diseases is increasingly growing. Among those sources, natural antioxidants from different plants (e.g., *Dirmophandra mollis* Benth, *Ruta graveolens* L., *Ginkgo biloba* L., *Vitis vinifera* L, *Thymus zygis*, *Croton argyrophyllus* Kunth, *Hordeum vulgare, Dalbergia ecastophyllum, Origanum vulgare* L.) are receiving particular attention [2,3,4,5,6,7]. 

Propolis (or bee glue) is a complex mixture of molecules obtained from several resinous secretions (e.g., resins, mucilage, gums, lattices, leaf buds) collected by worker-bees from different plant species [8]. Its composition is thus strongly influenced by the diversity of local flora, harvesting place and season, and by the genetics of the bees [9]. Briefly, these plant resinous secretions are mixed with salivary and enzymatic secretions of honey bees, producing a cementing material, used to close open cracks occurring in beehives. Propolis is used for sealing the spaces in beehives and to smooth their internal walls, providing antiseptic activity, as it protects the bees’ larvae and comb from microbial invasions [8]. 

Among several species, red propolis obtained from *Dalbergia ecastophyllum* (L.) Taub. (Fabaceae, [10]) shows various pharmacological properties of clinical interest, also being considered a functional food [11,12,13]. In particular, and due to its significant antioxidant activity [14,15,16], Brazilian red propolis has been proposed for dermal healing [17,18], and for the treatment of cancer cells [14,15,19,20], as an antimicrobial [16], antiproliferative [21,22], antihypertensive [23], and as a neuroprotective [11]. Several compounds have been identified in this resin, including the isoflavonoids formononetin, biochanin A, medicarpin, and pinocembrin [24,25]. Reported biological activities of these isolated compounds include the anti-inflammatory activities of biochanin A [26], pinocembrin [27], neovestitol, and vestitol [28], the antioxidant activities of formononetin [29], biochanin A [30], pinocembrin [31], the gastroprotective properties of biochanin A [32], the antifungal and dyslipidemic activities of formononetin [33,34], and the antimicrobial actions of neovestitol and vestitol [28].

Inhibitors of the HMG-CoA (3-hydroxy-3-methyl-glutaryl-coenzyme A) reductase play an active role in the treatment of hypercholesterolemia and have been found to play a protective role in several bacteria-associated diseases such as *Helicobacter pylori*-induced gastric ulcer. Association between *Helicobacter pylori* infection and dyslipidemia has been reported significantly [35]. Gastric ulcer is a chronic disease of high worldwide prevalence, associated to unexpected complications such as bleeding, perforation, and stenosis [36]. The imbalances of gastric mucosal defense mechanisms (prostaglandins, mucus, mucosal blood flow, bicarbonate, nitric oxide, and sulfhydryl compounds, and ATP sensitive K^+^ channels) against exposure to endogenous and exogenous factors (e.g., non-steroidal anti-inflammatory drugs (NSAIDs), pepsin, bile acids, alcohol, stress, and trauma, *Helicobacter pylori* infection, hemorrhagic shock, sepsis, and burns) promote the development of gastric ulcers [36,37].

It is also known that a long-term use of NSAID, and/or the presence of *Helicobacter pylori* infection, are the main causes of incidence and recurrence of gastric ulcers [38,39,40]. Furthermore, excessive alcohol consumption is an independent risk factor for the set-up of gastric ulcer, being thus a serious public health concern [41,42].

Using a rat model of inflammatory and neurogenic pain, without emotional or motor side effects, we have already demonstrated that hydroalcoholic extracts of red propolis (HERP) have significant anti-inflammatory and antinociceptive activities [43]. We have also described the anti-inflammatory activity of the dyslipidemic isoflavonoid formononetin [11,43]. In the present work, we proposed the in vivo characterization of the gastroprotective effects of HERP and formononetin, using an experimental rat model of gastric ulcer, as a suitable approach for phytomedical uses.

## 2. Materials and Methods

### 2.1. Drugs and Reagents

Alcian blue (Cas. No: 33864-99-2), carbenoxolone (Cas. No: 5697-56-3), cimetidine (Cas. No: 51481-61-9), formononetin (Cas. No: 485-72-3), indomethacin (Cas. No: 53-86-1), and omeprazole (Cas. No: 73590-58-6) were obtained from Sigma Chemical Co. (St. Louis, MO, USA). All other used reagents were of analytical grade. Test substances were dissolved in 0.9% NaCl solution containing 2% Tween 80 (polysorbate 80), while indomethacin was dissolved in 2% sodium bicarbonate, all from Sigma-Aldrich (Darmstadt, Germany). HERP and formononetin doses were based on our preliminary experiments [5].

### 2.2. Red Propolis Collection, Hydroalcoholic Extracts Preparation and Characterization

Red propolis was collected from species of Brejo Grande, Sergipe, Brazil (10°28′25″ S, 36°26′12″ W). The material was labeled, and stored in sterile and refrigerated containers. For the extraction, a volume of 12.5 mL of ethanol (70%) was used to extract 1 g of propolis samples using an ultrasound bath for 1 h, at room temperature as described by Pinheiro et al. [22]. After extraction, the obtained mixture was centrifuged, and the obtained supernatant evaporated under low pressure resulting in HERP, which was stored at refrigerated temperature until further use. The obtained extraction yield was 34% (w/w). Analysis of hydroalcoholic extracts of red propolis (HERP) was performed using HPLC as described by Cavendish et al. [43], and formononetin (ca. 2%) was confirmed as the major component.

### 2.3. Antioxidant Activity

The antioxidant activity of HERP was determined as the capacity of the antioxidants present in the sample to scavenge the stable radical 2 2-diphenyl-1-picrylhydrazyl (DPPH·), applying a previously described methodology [44,45]. A volume of 3 mL of extract, at concentrations between 2.5 and 15.0 mg/mL, was homogenized with 750 µL of DPPH solution (200 mM). After 15 min at room temperature, the spectrophotometric reading was done at 517 nm. Ethanol 70% was used as control and subject to the same procedure. Experiments were done in triplicate. To determine the % of free radical inhibition (%I), the following equation was used:

%I = 100 − (A_C_/A_A_) × 100

where A_C_ is the absorbance of the control and A_A_ is the absorbance of the sample. The mean inhibition index (IC50) was obtained from the %I values at each tested concentration by non-linear regression by computing the data in Prism GraphPad software (San Diego, CA, USA).

### 2.4. Microorganism

*Helicobacter pylori* strain ATCC 43504 was obtained from the Instituto Nacional de Controle de Qualidade em Saúde (INCQS—Fundação Oswaldo Cruz—Fiocruz/RJ, Brazil). The stock cultures were kept in Brucella broth at −20 °C until further use.

### 2.5. Animals

Wistar rats of both sexes, of 280–320 g mean weight, were received from the animal facilities of the Tiradentes University biotherium (Aracaju, Brazil). Animals were housed at 21 ± 2 °C in plastic cages, with free access to food (Purina^®^) and water ad libitum, under a 12-h light/12-h dark cycle. Twenty-four hours prior to the experiments, rats were provided with water ad libitum. All experiments and protocols were approved by the Institutional Ethics Committee (250608) of the Tiradentes University (Aracaju, Brazil) and were performed according to the EEC Directive of 1986, following the principles for laboratory animal use and care.

### 2.6. Antiulcerogenic Activity

#### 2.6.1. Ethanol-Induced Ulcers

These experiments were conducted according to the method described by Robert et al. [46]. Briefly, rats (*n* = 6/group) were fasted for 24 h and were then pre-treated orally with 50, 250, or 500 mg/kg HERP, 10 mg/kg formononetin, 100 mg/kg omeprazole, or vehicle (2% Tween 80, 10 mL/kg). Thirty minutes after treatment, all rats received 1 mL of absolute ethanol to induce gastric ulcers and were anaesthetized (3% halothane) and one hour later were euthanized by cervical dislocation. 

The stomachs of the animals were then removed and opened along the greater curvature. To remove gastric contents and blood clots, the stomachs were gently rinsed with water. Tissues images were recorded as described by Pinto et al. [47], and total lesion areas were measured (mm^2^). Stomach samples from 50, 250, and 500 mg/kg HERP, formononetin, omeprazole, and vehicle treated animals were cut into serial 5 µm thick sections and were stained with hematoxylin-eosin (HE) according to laboratorial techniques. 

Microscopic scores were determined for the interstitial edema and for the disruption of superficial regions of the gastric gland with epithelial cell loss Intensities of epithelial cell loss and interstitial edema were categorized as follows: absence (0); focal limited to the upper third (1); focal beyond the upper third (2); and diffuse in the upper third (3). Three histological sections for each animal (*n* = 6/group) were randomly analyzed (without previous knowledge of the treatments).

#### 2.6.2. Non-Steroidal Anti-Inflammatory Drug (NSAID)-Induced Ulcers

These experiments were adopted from the method described by Djahanguiri, with a few modifications [48]. Briefly, rats (*n* = 6/group) were pre-treated orally with HERP (50, 250, or 500 mg/kg), formononetin (10 mg/kg), cimetidine (100 mg/kg), or vehicle (2% Tween 80) for 30 min, and were then treated with indomethacin (100 mg/kg, p.o.) to induce gastric ulcers. Animals were euthanized by cervical dislocation six hours later after anesthesia with 3% halothane. Stomachs were then removed and opened for the scoring of ulcer index as follows: (i) score 1, standing for loss of mucosal folding, mucosal discoloration, edema, or hemorrhage; (ii) score 2, standing for less than 10 petechiae; and (iii) score 3, standing for more than 10 petechiae [49].

### 2.7. Determination of Gastric Juice Parameters Following Pyloric Ligature

For the determination of the gastric juice parameters, the methods described by Shay et al. were followed with some modifications [50]. Animals were anaesthetized with ketamine/xylazine (60 and 10 mg/kg, respectively, i.p.) after 24 h of fasting, followed by laparotomy and pylorus ligation. Rats (*n* = 6/group) were then treated intraduodenally with HERP (50, 250, or 500 mg/kg), formononetin (10 mg/kg), cimetidine (100 mg/kg), or vehicle (2% Tween 80). Four hours later, animals were anesthetized with halothane (3%) and euthanized by cervical dislocation. Abdomens were then opened, stomachs removed, and gastric contents collected and centrifuged for 10 min at 8000× *g* (25 °C). The pH and volume of the gastric juice values were measured and total acid secretions in total gastric juice supernatants determined by titration with 0.01 N NaOH solution using phenolphthalein as indicator.

### 2.8. Determination of Gastric Mucus Contents

For the determination of the mucus contents the methods described by Sun et al. were followed, with some modifications [51]. Animals were anaesthetized with ketamine/xylazine (60 and 10 mg/kg, respectively, i.p.) after 24 h of fasting, followed by laparotomy and pylorus ligation. Rats (*n* = 6/group) were immediately treated intraduodenally with HERP (50, 250, or 500 mg/kg), formononetin (10 mg/kg), carbenoxolone (200 mg/kg), or vehicle (2% Tween 80). Four hours later, animals were anesthetized with halothane (3%) followed euthanasia by cervical dislocation. The removed stomach contents were immersed in 10 mL of solution containing 0.16-M sucrose, 0.02% Alcian blue, and 0.05-M sodium acetate (pH 5.8), and were kept at room temperature for 24 h. Alcian blue binding extracts were then centrifuged at 3000× *g* for 10 min and absorbances of supernatants were measured using spectrophotometry at 620 nm. Free gastric mucus contents were calculated from quantities of bound Alcian blue (mg/g tissue).

### 2.9. Agar-Well Diffusion Assays

Antibacterial activity was investigated using a modified agar-well diffusion method [52]. Wells of 8 mm in diameter were sub-cultured with blood agar (Mueller-Hinton agar with 10% sheep blood) plates and inoculated with *Helicobacter pylori* suspensions of 6 × 10^8^ CFU/mL (McFarland turbidity standard 0.5). Wells were then filled with 80 μL of 100 mg/mL HERP and tetracycline (0.03 mg/mL) was used as a standard compound. Plates were stored for 30 min at room temperature and then incubated for another 48 h at 37 °C under microaerophilic conditions. Subsequently, growth inhibition halos were measured using a digital pachymeter. The size of the inhibitory zones (diameter) was measured in triplicate and mean values of ≥08 mm considered active.

### 2.10. Statistical Analysis

Data are presented as the mean ± standard error of the mean (SEM) of n animals per group. Differences between the treated groups were identified using the one-way analysis of variance (ANOVA) and Bonferroni’s test. Changes in microscopy parameters of indomethacin-induced ulcers (scores) were analyzed using Kruskal–Wallis test followed by Dunn’s test. Differences were considered significant when *p* < 0.05.

## 3. Results

From the in vitro antioxidant activity test against free radical DPPH, an IC50 of 294 µg/mL was recorded for HERP. This result demonstrates the antioxidant potential of the variety of propolis used in this study. The antioxidant activity of the extract may be related to its ability to inhibit ethanol damage on the gastric mucosa. Ethanol is metabolized in the body, releasing superoxide anion and hydroperoxides. Such free radicals are known to be involved in the mechanism of acute and chronic ulceration. The sequestration of these chemical entities plays an important role in the healing process of gastric injury [53].

Free radicals are chemical entities capable of causing irreversible damage to the cell [4,54]. The antioxidant process includes the scavenging of these radicals (preventing their propagation), enzymatic hydrolysis of the ester linkage to remove peroxidized fatty acids from lipids, scavenging of transition metal ions, and reduction of peroxide catalyst enzymes. The pathogenesis of peptic ulcer is associated with endogenous and exogenous factors. Ethanol causes oxidative stress, which can happen by decreasing the release of nitric oxide, which has an important role in the metabolism of free radicals [55].

**Figure 1:**
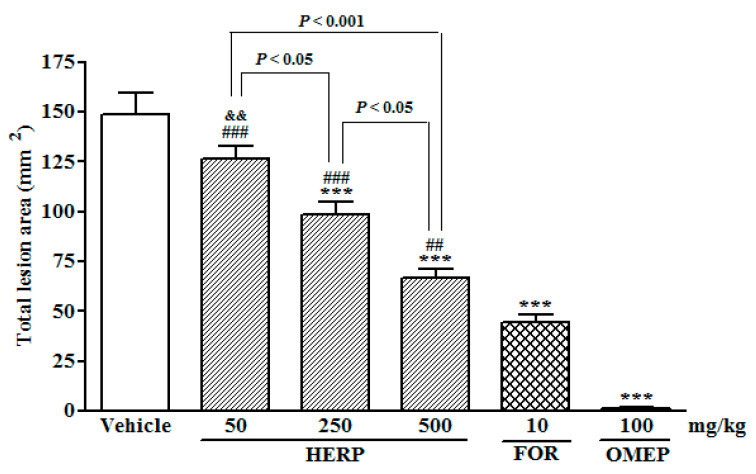
Effects of hydroalcoholic extracts of red propolis (HERP) and formononetin (FOR) on the ethanol-induced gastric damage; rats were pre-treated with a solution of 2% Tween 80 (vehicle), HERP (50–500 mg/kg), formononetin (10 mg/kg), or omeprazole (OMEP, 100 mg/kg) for 30 min prior to one hour treatment with absolute ethanol (1 mL). Total lesion areas were then determined and are presented as the means ± standard errors of the mean (SEM; *n* = 6/group); ANOVA followed by Bonferroni’s test; *** *p* < 0.001 vs. vehicle group; ^##^ *p* < 0.01 and ^###^ *p* < 0.001 vs. OMEP group; ^&&^ *p* < 0.01 vs. FOR group.

**Figure 2:**
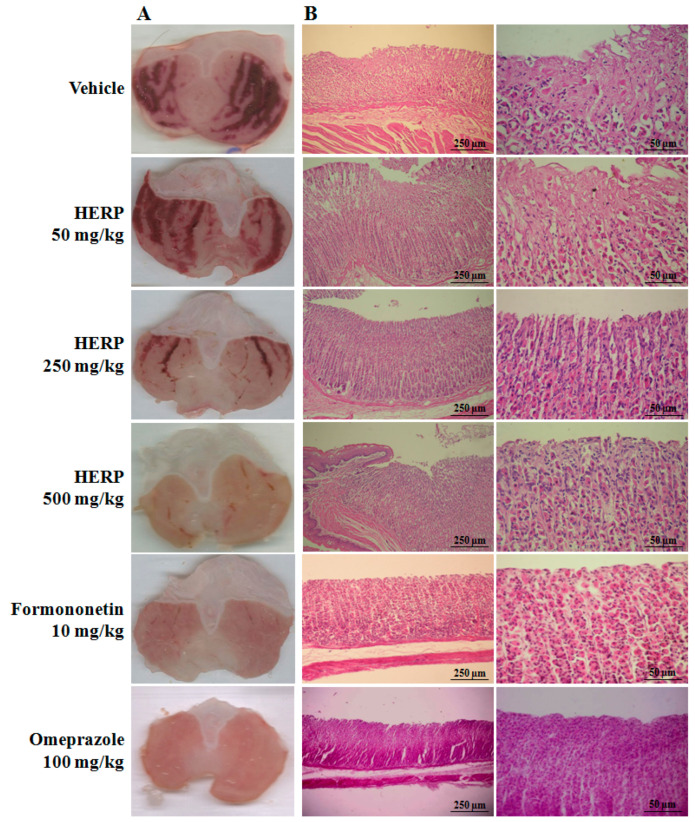
(**A**) Macroscopic effects of HERP, formononetin, and omeprazole in ethanol-induced gastric ulcers; (**B**) hematoxylin/eosin-stained histological sections of gastric mucosa specimens from ethanol treated rats; representative photomicrographs were generated from the same areas and were quantified (Table 1).

Following the induction of ulcers using ethanol, pre-treatments with the HERP at different doses (50, 250, or 500 mg/kg) inhibited the total lesion areas in a dose dependently fashion, when compared to the group treated with Tween 80 (2%) solution (*p* < 0.001; Figure 1). Formononetin (FOR, 10 mg/kg) and omeprazole (OMEP, 100 mg/kg) also reduced the areas of the total lesions significantly (*p* < 0.001) (Figure 1). Table 1 depicts the quantification effects of HERP and formononetin on ethanol-induced damage of gastric mucosa. Histopathological analyses of gastric mucosa after the administration of absolute ethanol are shown in Figure 2.

The oral administration of absolute ethanol administration (vehicle-treated group) promoted microscopic damage, with a pronounced edema of the submucosa and a consistent epithelial cell loss, and presence of inflammatory cells. Ethanol-induced gastric ulcer resulted from the disruption of the vascular endothelium, increase of vascular permeability, formation of edema, and promotion of epithelial lifting. The gastric tissue of the animals treated with HERP (250 and 500 mg/kg, p.o.) showed less mucosal damage than those that were treated with the vehicle. The total lesion area was reduced in a concentration dependent manner, i.e., the increase of the HERP dose promoted a significant reduction of the total lesion area. Pre-treatment with formononetin and omeprazole also inhibited the formation of ethanol-induced lesions (vehicle group). Omeprazole is a proton-pump inhibitor thus it reduces the acid produced in the stomach. It is commonly used to promote the healing of erosive esophagitis and for the treatment of other gastrointestinal conditions (e.g., gastroesophageal reflux disease, peptic ulcer). Omeprazole treatment (OMEP, 100 mg/kg) for 30 min resulted in a total lesion area close to zero (mm^2^).

The macroscopic effects of HERP shown in Figure 2 clearly demonstrate a significant tissue recovery in the animals treated with HERP (250 and 500 mg/kg, *p* < 0.05) and formononetin (10 mg/kg, *p* < 0.001), against the positive control (vehicle). Pre-treatments with HERP doses (250 and 500 mg/kg, *p* < 0.05) and formononetin (10 mg/kg, *p* < 0.001) significantly reduced ulcer indices of indomethacin-induced ulcers in comparison with the those of the vehicle-treated group (Table 2). Cimetidine (100 mg/kg) also significantly reduced ulcer indices (*p* < 0.01, Table 2). Indomethacin is an indol derivative that is the first-choice approach for gastric-ulcer induction in laboratory. Cimetidine belongs to the histamine H2 receptor antagonists and acts by inhibiting the production of acid in the stomach. It is commonly used to treat and prevent certain types of stomach ulcer. 

The treatment with 50 and 250 mg/kg HERP in the pylorus ligature model of gastric secretion significantly reduced secretion volumes (*p* < 0.01) and increased H^+^ concentrations (50 mg/kg, *p* < 0.01) (Table 3). Similarly, treatments with formononetin (10 mg/kg) significantly reduced secretion volumes (*p* < 0.01). However, HERP and formononetin treatments both did not succeed in significantly increasing the pH values compared to those in the group treated with the vehicle. In contrast, cimetidine affected all gastric juice parameters significantly (*p* < 0.01).

Compared to the vehicle treated controls (Table 4), the production of mucus was significantly increased with HERP (500 mg/kg) treatment (*p* < 0.05). Treatments with formononetin (FOR, 10 mg/kg) and carbenoxolone (200 mg/kg) also increased the production of mucus (*p* < 0.05 and *p* < 0.001, respectively, when compared to the control).

In assessments of anti-*H. pylori* activity, HERP treatments reduced the diameter of the inhibitory zone to 13.0 ± 2.0 mm at 100 mg/mL, whereas inhibition halos were 35.0 ± 0.5 mm in the presence of the standard tetracycline (0.03 mg/mL).

## 4. Discussion

Propolis is a product obtained from honey bee hives, characterized chiefly by a beeswax and a resin (obtained from apical buds, young leaves and exudates) [56]. Red propolis is ranked as the second most produced type of Brazilian propolis. Its characteristics (e.g., texture, odor, color) vary according to the source and type, but also harvesting season. The interest in Brazilian red propolis is attributed to its several beneficial effects for a range of health conditions, due to the presence of phytoestrogens. Phytoestrogens are naturally occurring compounds with a chemical structure similar to 17-β-estradiol, the mammalian estrogen. These compounds show the capacity to bind to estrogen receptors, either as agonists or antagonist, to promote, respectively, estrogenic or antiestrogenic activity [57]. They have been recommended for the treatment of estrogen-related diseases, as happens with dyslipidemia resulting from the high levels of estrogen in post-menopause women. On the other hand, during physiologic menstrual cycle bicarbonate secretion increases with estrogenic levels, which may contribute to reduce the risk of gastric ulcer. 

**Figure 3:**
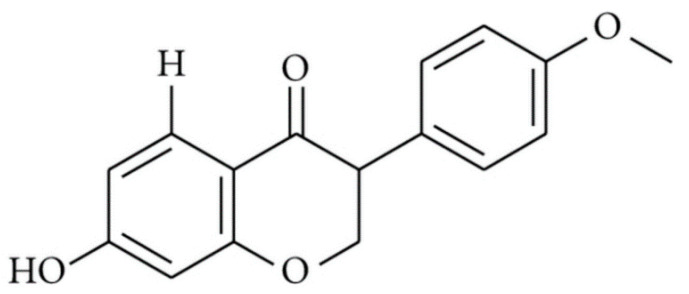
Chemical structure of formononetin (C16H12O4, molecular weight 268.26 g/mol).

In our study, two rat ulcer models were used (ethanol-induced and indomethacin-induced ulcers) and tested for the potential of hydroalcoholic extracts of red propolis (HERP), and its isoflavonoid formononetin, for gastric protection. The phytoestrogen formononetin is an O-methylated isoflavone (Figure 3) that was found to be present in hydroalcoholic extract of Brazilian red propolis. 

Our results demonstrate that HERP in the tested concentrations of 50, 250, and 500 mg/kg exhibited acute gastroprotective effects against ethanol- and indomethacin-induced ulcers, in a concentration dependent manner, with the highest dose increasing the mucus production significantly (Table 4). HERP and its major isoflavonoid formononetin (10 mg/Kg) decreased gastric secretion volumes and increased mucus production in the present pylorus ligature model, while HERP depicted anti-*H. pylori* activity. Oral administration of HERP decreased the total lesion areas (Figure 1) induced by absolute ethanol in a dose dependent fashion, and reduced the associated macroscopic and microscopic damage to mucosa (Figure 2), at doses which were considered safe, according to the toxicological study described by Silva et al. [58]. Similar reductions were also observed after formononetin treatment when compared to the vehicle-treated animals, which showed severe ulceration of gastric mucosa (vehicle group).

Ethanol has been previously used to induce ulcers in rodents, leading to gastric mucosal injury through the direct and indirect effects of reactive oxygen species (ROS) and cytokines [34]. Moreover, this process is reportedly mediated by activated neutrophils, and is associated with cellular lipid and protein peroxidation [59]. However, several studies have shown that natural products with antioxidant activity protect gastric mucosa against damage [60,61,62,63]. The advantages of HERP for human use have been extensively discussed in the literature, relying on the many identified beneficial effects, mostly attributed to its antioxidant properties [5,64,65,66,67]. Various phenolic antioxidants, such as flavonoids, coumarins, tannins, procyanidins, and xanthenes, act as radical scavengers, neutralizing ROS, inhibiting lipid peroxidation and other free radical-mediated pathologies, in a dose-dependent fashion. Plants enriched with antioxidants, such as isoflavonoid, may thus be considered as an alternative approach for the management and treatment of the chronic diseases related to oxidative stress. Among flavonoids present in HERP, biochanin A has demonstrated gastroprotective effects by a strong induction of superoxide dismutase (SOD) and nitric oxide enzymes, and reduced release of malondialdehyde [32]. Asif et al. have reported a strong correlation between the antioxidant and antiglycation activities of biochanin A present in organic fractions of *Hordeum vulgare* [6].

The therapeutic potential of biochanin-A against myocardial infarction has also been proposed, and attributed to its capacity in compromising lipid peroxidation. Male Wistar rats were first subjected to isoproterenol-induced myocardial infarction [68], resulting in a significant increase of creatine kinase-MB and cardiac troponin, serum glutamic oxaloacetic transaminase, serum glutamic pyruvic transaminase, and lactate dehydrogenase, and reduction of the activity of antioxidant enzymes (i.e., SOD, catalase, glutathione peroxidase, glutathione-S-transferase, and glutathione reductase) in the heart. Such antioxidant enzymes catalyze reactions to counterbalance free radicals and ROS, thus forming endogenous defense mechanisms. The authors reported the cardioprotective effects of biochanin-A by modulation of lipid peroxidation, through enhancing antioxidants and detoxifying enzyme systems [68]. The effect of biochanin-A on a high-fat diet-induced hyperlipidemia in mice has been studied by Xue et al. [69]. The authors have demonstrated the capacity of biochanin A in decreasing the LDL-cholesterol in about 85% and total cholesterol ca. 39% in a moderate dose, increasing the activity of lipoprotein lipase in 96% and of hepatic triglyceride lipase in 78%. The results also showed the increase of fecal lipid levels and decrease of epididymal fat index in hyperlipidemic mice in comparison to the control mice. Jia et al. demonstrated that formononetin attenuates hydrogen peroxide-induced apoptosis and nuclear factor-kappa B (NF-κB) activation in retinal ganglion cells, exposed to oxidative stress over a period of 24 h [70]. 

The abovementioned beneficial effects of flavonoids (biochanin-A, formononetin) identified in HERP, substantiate the rationale for exploiting additional biological activities related to their antioxidant properties. Fukai et al. anticipated the potential use of these compounds as chemopreventive agents for peptic ulcer or gastric cancer in *Helicobacter pylori*-infected individuals [71]. 

*Helicobacter pylori* infections are associated with dyslipidemia. Epidemiologic and clinical data suggest that such an infection can be a contributing factor in the progression of atherosclerosis [35]. In 2010, Satoh et al. reported that *Helicobacter pylori* infection was significantly associated with high LDL-cholesteremia and low HDL-cholesteremia in Japanese male subjects [72]. The association of this infection with metabolic syndrome in the Japanese population, has also been reported in the past [73]. Thus, treating *Helicobacter pylori* infections and protecting gastric mucosa from ulceration may have a synergistic effect in prophylaxis of dyslipidemia as well. 

From our results, the histological analyses obtained from ethanol-induced ulcers show that HERP and formononetin attenuate lymphocytic infiltration, suggesting immunomodulatory effects on the release of cytokines (Figure 2). Furthermore, bioactive fractions of geopropolis that were collected from the same region reportedly decreased neutrophil migration and reduced NO-related inflammatory interactions between leukocytes and endothelial cells [74].

Numerous studies have demonstrated enhanced immune responses in the presence of propolis [75,76,77,78]. Moreover, the isoflavonoid biochanin A inhibited lipopolysaccharide (LPS)-induced activation of microglia, and production of tumor necrosis factor (TNF)-α, NO, and SOD in mesencephalic neuron-glia cultures and microglia-enriched cultures [79]. Formononetin also inhibited TNF-α and interleukin (IL)-6 expression, and improved SOD activity in traumatic brain injury [29] and LPS-induced acute lung injury models [80]. These studies suggest that the gastroprotective properties of HERP are mediated, at least partially, by formononetin.

NSAIDs are the most commonly used treatment for inflammatory diseases, but are proven to cause gastric and duodenal ulcers in rats and humans [81]. The potent NSAID indomethacin causes gastric legions by inhibiting cyclo-oxygenase (COX), resulting in decreased prostaglandin synthesis [82]. The ensuing injury is characterized by reduced bicarbonate and mucus secretion and blood flow, increased acid back-diffusion, and inhibition of repair. In the present study, HERP and its flavonoid formononetin inhibited the formation of indomethacin-induced gastric lesions. Flavonoids were previously shown to inhibit COX-1 and COX-2 enzymes [83], and the isoflavonoids genistein and daidzein significantly reduced COX activity [84]. Hence, the present observations of increased mucus production in the presence of HERP or formononetin were attributed to COX inhibition. 

Flavonoid treatments reportedly increased the production of mucus and bicarbonate, and affected proton pump activities in parietal cells [85]. Hence, the present effects of HERP likely reflect formononetin contents. Hajrezaie et al. showed that biochanin A increases mucus secretion in ethanol-induced ulcer models [32], and De Barros et al. showed that quercitrin and afzelin increase the secretion of mucus and reduce H^+^- and K^+^-ATPase activity in vitro [86].

In further experiments, HERP and formononetin decreased gastric secretion volumes after pylorus ligature. As HERP and formononetin treatments were administered via a intraduodenal route, systemic effects are likely not to be related to the local neutralization of gastric acid or physical barriers. Moreover, physiological gastric acid secretion by parietal cells is activated by several stimuli. These include acetylcholine, which acts directly via M_3_ receptors and indirectly by M_2_ and M_4_ receptors, and is associated with the inhibition of somatostatin secretion, histamine, which acts directly via H_2_ receptors, and gastrin, which predominantly acts indirectly via cholecystokinin-2 (CCK-2) receptors on enterochromaffin-like cells and is associated with histamine release [87]. Flavonoids have been shown to induce the expression of acetylcholinesterase, which hydrolyzes acetylcholine in cultured osteoblast synapses [88]. Additionally, flavonoids reportedly increase mucosal prostaglandin levels and reduce histamine production by mast cells by inhibiting histidine decarboxylase [89].

Herein, we showed increased H^+^ concentrations following treatments with HERP, likely reflecting the presence of acid phenolic compounds [16]. However, no significant changes in pH were observed under these conditions, whereas cimetidine treatment modified all biochemical parameters of gastric mucosa, suggesting distinct mechanisms of action. Similarly, formononetin treatments reduced gastric secretion volumes, but had no significant effects on pH or total acid secretion, indicating that changes in gastric acid secretions do not play an important role in the gastroprotective mechanisms of this compound. *H. pylori* is associated with various gastric diseases and is a frequently encountered human pathogen [90]. In our study, we demonstrated that HERP treatment (100 mg/mL) inhibited the growth of *H. pylori* in vitro, highlighted by the identification of an inhibitory zone of 13.0 ± 2.0 mm, in comparison to the 35.0 ± 0.5 mm inhibition halos in the presence of standard tetracycline (0.03 mg/mL). The anti-*H. Pylori* effect has been demonstrated for several propolis varieties. Using the same agar-well diffusion method, Boyanova et al. (2003) reported the increase of mean diameters of *H. pylori* growth inhibition from 17.8, 21.2, 28.2 mm when treated, respectively, by 30, 60, and 90 mL of ethanolic propolis extract (30%), whereas the treatment with of ethanol alone (30 mL) resulted in a mean diameter of 8.5 mm [91]. More recently, Baltas et al. (2016) reported inhibition diameters ranging from 31.0 to 47.0 mm from 15 different ethanolic propolis extracts tested at 75 μg/mL [92]. The authors found a significant positive correlation between the reported anti-*H. pylori* activity of the extracts and the total phenolic content.

## 5. Conclusions

In this work, we reported the gastroprotective effect of HERP, and its predominant isoflavonoid formononetin, known for its hypolipidemia effects. Our results demonstrated that treating animals with HERP resulted in the reduction of mucosal damage, in particular with doses as high as 200 and 500 mg/Kg (p.o.), with a significant reduction in the loss of epithelial cells and on edema. The macroscopic effects of sample-treated rats corroborate these results. The treatment with HERP increased H^+^ concentration, as well as the mucus production, both contributing to enhance the protection of gastrointestinal mucosa, and to reducing the aggressive effects in gastric mucosa, thus reducing the risk of ulcers. The antioxidant activity of HERP was also shown, reporting a IC50 of 294 µg/mL. Together with the prophylactic role in reducing the lipid accumulation, formononetin was shown to have a prominent role in increased mucus production. HERP can be further exploited as an alternative phytomedicine for the treatment of ulcers with hypolipidemic profile. HERP and formononetin have recognized gastroprotective properties, as they significantly inhibit the development of acute ulcers in the presence of ethanol and indomethacin, ameliorated inflammatory cell infiltration and edema, reducing gastric secretion volumes, and increasing gastric mucus contents.

## Figures and Tables

**Table 1:** Effects of HERP and formononetin on ethanol-induced microscopic damage in gastric mucosa.

Treatment (p.o.)	Dose (mg/kg)	Epithelial Cell Loss (Score 0–3)	Edema (Score 0–3)
Vehicle	-	3.0 (2.0–3.0)	3.0 (2.0–3.0)
HERP	50	3.0 (2.0–3.0) ^###^	0.0 (0.0–2.0) ***
	250	1.0 (0.0–2.0) ***	0.0 (0.0–1.0) ***
	500	1.0 (0.0–2.0) ***	0.0 (0.0–1.0) ***
Formononetin	10	0.0 (0.0–1.0) ***	0.0 (0.0–1.0) ***
Omeprazole	100	0.0 (0.0–1.0) ***	0.0 (0.0–1.0) ***

Data are presented as medians with minimum and maximal scores in brackets; differences were identified using Kruskal–Wallis followed by Dunn’s test; *** *p* < 0.001 vs. vehicle group; and ^###^ *p* < 0.001 vs. HERP groups at 250 and 500 mg/kg; three histological sections from each animal, *n* = 6/group.

**Table 2:** Effects of HERP and formononetin on indomethacin-induced ulcers in rats.

Treatment (p.o.)	Dose (mg/kg)	Ulcer Index	Inhibition (%)
Vehicle	-	2.29 ± 0.18	-
HERP	50	1.86 ± 0.14 ^##^	18.78
	250	0.29 ± 0.18 *	87.34
	500	0.00 ± 0.00 ***	100.00
Formononetin	10	0.00 ± 0.00 ***^&&^	100.00
Cimetidine	100	0.29 ± 0.29 **^&^	87.34

Results are presented as means ± SEM (*n* = 6/group). Differences were identified using Kruskal–Wallis test followed by Dunn’s test; * *p* < 0.05, ** *p* < 0.01, and *** *p* < 0.001 vs. vehicle group; ^##^ *p* < 0.01 vs. 500-mg/kg HERP group; ^&^ *p* < 0.05 and ^&&^ *p* < 0.01 vs. 50-mg/kg HERP group.

**Table 3:** Effects of intraduodenal treatments with HERP and formononetin on the biochemical parameters of gastric juice collected from the pylorus ligature rats.

Treatment	Dose (mg/kg)	Volume (mL)	pH	[H^+^]mEq/L/4 h
Vehicle	-	1.20 ± 0.04	3.36 ± 0.05	46.8 ± 2.85
HERP	50	0.70 ± 0.08 ***^#^	3.40 ± 0.12	65.9 ± 2.69 **^###^
	250	0.80 ± 0.08 **	3.40 ± 0.12	56.0 ± 3.35 ^##^
	500	1.00 ± 0.08	3.30 ± 0.00	36.3 ± 3.55
Formononetin	10	0.82 ± 0.05 **	3.14 ± 0.14	42.7 ± 3.78
Cimetidine	100	0.80 ± 0.04 **	6.10 ± 0.45 ***	27.5 ± 3.14 **

Results are presented as means ± SEM (*n* = 6/group) and differences between treatment groups were identified using ANOVA followed by Bonferroni’s test; ** *p* < 0.01 and *** *p* < 0.001 vs. vehicle group; ^#^ *p* < 0.05, ^##^ *p* < 0.01, and ^###^ *p* < 0.001 vs. 500-mg/kg HERP group.

**Table 4:** Effects of intraduodenal treatments with HERP and formononetin on Alcian blue binding to free gastric mucus from pylorus ligatures in rats.

Treatment	Dose (mg/kg)	Alcian Blue Bound (mg/g Tissue)
Vehicle	-	1.14 ± 0.03
HERP	50	1.21 ± 0.02
	250	1.25 ± 0.02
	500	1.30 ± 0.02 *
Formononetin	10	1.34 ± 0.07 *
Carbenoxolone	200	1.53 ± 0.03 ***

Results are presented as means ± SEM (*n* = 6/group) and differences were identified using ANOVA followed by the Bonferroni’s test; * *p* < 0.05 and *** *p* < 0.001 vs. vehicle group.

## References

[1] Ito F., Sono Y., Ito T. (2019). Measurement and Clinical Significance of Lipid Peroxidation as a Biomarker of Oxidative Stress: Oxidative Stress in Diabetes, Atherosclerosis, and Chronic Inflammation. Antioxidants.

[2] Carbone C., Martins-Gomes C., Caddeo C., Silva A.M., Musumeci T., Pignatello R., Puglisi G., Souto E.B. (2018). Mediterranean essential oils as precious matrix components and active ingredients of lipid nanoparticles. Int. J. Pharm..

[3] Cefali L.C., Ataide J., Fernandes A.R., Sanchez-Lopez E., Sousa I., Figueiredo M.C., Ruiz A., Foglio M., Mazzola P.G., Souto E.B. (2019). Evaluation of In Vitro Solar Protection Factor (SPF), Antioxidant Activity, and Cell Viability of Mixed Vegetable Extracts from Dirmophandra mollis Benth, *Ginkgo biloba* L., *Ruta graveolens* L., and *Vitis vinífera* L.. Plants.

[4] Silva A.M., Martins-Gomes C., Souto E.B., Schäfer J., Dos Santos J.A., Bunzel M., Nunes F.M. (2020). Thymus zygis subsp. zygis an Endemic Portuguese Plant: Phytochemical Profiling, Antioxidant, Anti-Proliferative and Anti-Inflammatory Activities. Antioxidants.

[5] Carvalho F.M.D.A.D., Schneider J.K., De Jesus C.V.F., Andrade L., Amaral R.G., David J.M., Krause L.C., Severino P., Soares C., Caramão E.B. (2020). Brazilian Red Propolis: Extracts Production, Physicochemical Characterization, and Cytotoxicity Profile for Antitumor Activity. Biomolecules.

[6] Asif A., Zeeshan N., Mehmood S. (2020). Antioxidant and antiglycation activities of traditional plants and identification of bioactive compounds from extracts of Hordeum vulgare by LC–MS and GC–MS. J. Food Biochem..

[7] Souto E.B., Severino P., Marques C., Andrade L.N., Durazzo A., Lucarini M., Atanasov A.G., El Maimouni S., Novellino E., Santini A. (2020). *Croton argyrophyllus* Kunth Essential Oil-Loaded Solid Lipid Nanoparticles: Evaluation of Release Profile, Antioxidant Activity and Cytotoxicity in a Neuroblastoma Cell Line. Sustainability.

[8] Anjum S.I., Ullah A., Khan K.A., Attaullah M., Khan H., Ali H., Bashir M.A., Tahir M., Ansari M.J., Ghramh H.A. (2019). Composition and functional properties of propolis (bee glue): A review. Saudi J. Boil. Sci..

[9] Santos L.M., Da Fonseca M.S., Sokolonski A.R., Deegan K.R., Araújo R.P.C., Umsza-Guez M.A., Barbosa J.D.V., Portela R.D., Machado B.A.S. (2019). Propolis: Types, composition, biological activities, and veterinary product patent prospecting. J. Sci. Food Agric..

[10] Daugsch A., Moraes C.S., Fort P., Park Y.K. (2008). Brazilian Red Propolis—Chemical Composition and Botanical Origin. Evid. Based Complement. Altern. Med..

[11] Barbosa R.A., Nunes T.L.G.M., Nunes T.L.G.M., Da Paixão A.O., Neto R.B., Moura S., Júnior R.L.C.A., Cândido E.A.F., Padilha F.F., Quintans J.S. (2015). Hydroalcoholic extract of red propolis promotes functional recovery and axon repair after sciatic nerve injury in rats. Pharm. Boil..

[12] Batista C., Alves A., Queiroz L., Lima B., Filho R., Araújo A., Júnior R.D.A., Cardoso J. (2018). The photoprotective and anti-inflammatory activity of red propolis extract in rats. J. Photochem. Photobiol. B Boil..

[13] Cavalcante D.R.R., De Oliveira P.S., Góis S.M., Soares A.F., Cardoso J.C., Padilha F.F., Júnior R.L.C.D.A. (2011). Effect of green propolis on oral epithelial dysplasia in rats. Braz. J. Otorhinolaryngol..

[14] Frozza C.O.D.S., Garcia C.S.C., Gambato G., De Souza M.D.O., Salvador M., Moura S., Padilha F.F., Seixas F.K., Collares T., Borsuk S. (2013). Chemical characterization, antioxidant and cytotoxic activities of Brazilian red propolis. Food Chem. Toxicol..

[15] De Mendonça I.C.G., Porto I.C.C.D.M., Nascimento T.G.D., De Souza N.S., Oliveira J.M.D.S., Arruda R.E.D.S., Mousinho K.C., Santos A.F., Júnior I.D.B., Parolia A. (2015). Brazilian red propolis: Phytochemical screening, antioxidant activity and effect against cancer cells. BMC Complement. Altern. Med..

[16] Righi A.A., Alves T.R., Negri G., Marques L.M., Breyer H., Salatino A. (2011). Brazilian red propolis: Unreported substances, antioxidant and antimicrobial activities. J. Sci. Food Agric..

[17] Júnior R.L.A., Barreto A.L.S., Pires J.A., Reis F.P., Lima S.O., Ribeiro M., Cardoso J.C. (2009). Effect of Bovine Type-I Collagen-Based Films Containing Red Propolis on Dermal Wound Healing in Rodent Model. Int. J. Morphol..

[18] De Almeida E.B., Cardoso J.C., De Lima A.K., De Oliveira N.L., De Pontes-Filho N.T., Lima S.O., Souza I.C.L., De Albuquerque-Júnior R.L.C. (2013). The incorporation of Brazilian propolis into collagen-based dressing films improves dermal burn healing. J. Ethnopharmacol..

[19] Awale S., Li F., Onozuka H., Esumi H., Tezuka Y., Kadota S. (2008). Constituents of Brazilian red propolis and their preferential cytotoxic activity against human pancreatic PANC-1 cancer cell line in nutrient-deprived condition. Bioorg. Med. Chem..

[20] Ribeiro D.R., Ângela V.F.A., Dos Santos E.P., Padilha F.F., Gomes M.Z., Rabelo A.S., Cardoso J.C., Massarioli A.P., De Alencar S.M., De Albuquerque-Júnior R.L.C. (2015). Inhibition of DMBA-induced Oral Squamous Cells Carcinoma Growth by Brazilian Red Propolis in Rodent Model. Basic Clin. Pharmacol. Toxicol..

[21] Frozza C.O.D.S., Ribeiro T.D.S., Gambato G., Menti C., Moura S., Pinto P.M., Staats C.C., Padilha F.F., Begnini K.R., De Leon P.M.M. (2014). Proteomic analysis identifies differentially expressed proteins after red propolis treatment in Hep-2 cells. Food Chem. Toxicol..

[22] Pinheiro K.S., Ribeiro D.R., Alves A.V.F., Pereira-Filho R.N., De Oliveira C.R., Cardoso J.C., Lima S.O., Reis F.P., Júnior R.L.A. (2014). Modulatory activity of brazilian red propolis on chemically induced dermal carcinogenesis. Acta Cir. Bras..

[23] Teles F., Da Silva T.M., Júnior F.P.D.C., Honorato V.H., Costa H.D.O., Barbosa A.P.F., De Oliveira S.G., Porfírio Z., Libório A.B., Borges R.L. (2015). Brazilian Red Propolis Attenuates Hypertension and Renal Damage in 5/6 Renal Ablation Model. PLoS ONE.

[24] López B.G.-C., Schmidt E.M., Eberlin M.N., Sawaya A.C.H.F. (2014). Phytochemical markers of different types of red propolis. Food Chem..

[25] Trusheva B., Popova M., Bankova V., Simova S., Marcucci M.C., Miorin P.L., Pasin F.D.R., Tsvetkova I. (2006). Bioactive Constituents of Brazilian Red Propolis. Evid. Based Complement. Altern. Med..

[26] Wang W., Tang L., Li Y., Wang Y. (2015). Biochanin A protects against focal cerebral ischemia/reperfusion in rats via inhibition of p38-mediated inflammatory responses. J. Neurol. Sci..

[27] Zhou L.-T., Wang K.-J., Li L., Li H., Geng M. (2015). Pinocembrin inhibits lipopolysaccharide-induced inflammatory mediators production in BV2 microglial cells through suppression of PI3K/Akt/NF-κB pathway. Eur. J. Pharmacol..

[28] Bueno-Silva B., De Alencar S.M., Koo H., Ikegaki M., Da Silva G.V.J., Napimoga M.H., Rosalen P.L. (2013). Anti-Inflammatory and Antimicrobial Evaluation of Neovestitol and Vestitol Isolated from Brazilian Red Propolis. J. Agric. Food Chem..

[29] Li Z., Dong X., Zhang J., Zeng G., Zhao H., Liu Y., Qiu R., Mo L., Ye Y. (2014). Formononetin protects TBI rats against neurological lesions and the underlying mechanism. J. Neurol. Sci..

[30] Wang J., He C., Wu W.-Y., Chen F., Wu Y.-Y., Li W., Chen H.-Q., Yin Y.-Y. (2015). Biochanin A protects dopaminergic neurons against lipopolysaccharide-induced damage and oxidative stress in a rat model of Parkinson’s disease. Pharmacol. Biochem. Behav..

[31] Saad M.A., Abdelsalam R.M., Kenawy S.A., Attia A.S. (2015). Pinocembrin attenuates hippocampal inflammation, oxidative perturbations and apoptosis in a rat model of global cerebral ischemia reperfusion. Pharmacol. Rep..

[32] Hajrezaie M., Salehen N., Karimian H., Zahedifard M., Shams K., Al Batran R., Majid N.A., Khalifa S.A.M., Ali H.M., El-Seedi H. (2015). Biochanin A Gastroprotective Effects in Ethanol-Induced Gastric Mucosal Ulceration in Rats. PLoS ONE.

[33] Das Neves M.V.M., Da Silva T.M.S., Lima E.D.O., Da Cunha E.V.L., Oliveira E.J. (2016). Isoflavone formononetin from red propolis acts as a fungicide against Candida sp.. Braz. J. Microbiol..

[34] Abdel-Salam O.M.E., Czimmer J., Debreceni A., Szolcsányi J., Mózsik G. (2001). Gastric mucosal integrity: Gastric mucosal blood flow and microcirculation. An overview. J. Physiol..

[35] Kim T.J., Lee H., Kang M., Kim J.E., Choi Y.-H., Min Y.W., Min B.-H., Lee J.H., Son H.J., Rhee P.-L. (2016). Helicobacter pylori is associated with dyslipidemia but not with other risk factors of cardiovascular disease. Sci. Rep..

[36] Gao H., Li L., Zhang C., Tu J., Geng X., Wang J., Zhou X., Jing J., Pan W. (2020). Comparison of efficacy of pharmacological therapies for gastric endoscopic submucosal dissection-induced ulcers: A systematic review and network meta-analysis. Expert Rev. Gastroenterol. Hepatol..

[37] Milivojevic V., Milosavljevic T. (2020). Burden of Gastroduodenal Diseases from the Global Perspective. Curr. Treat. Options Gastroenterol..

[38] Takahashi M., Katayama Y., Takada H., Kuwayama H., Terano A. (2000). The effect of NSAIDs and a COX-2 specific inhibitor on Helicobacter pylori-induced PGE2 and HGF in human gastric fibroblasts. Aliment. Pharmacol. Ther..

[39] Tytgat G.N. (2011). Etiopathogenetic Principles and Peptic Ulcer Disease Classification. Dig. Dis..

[40] Venerito M., Goni E., Malfertheiner P. (2016). Helicobacter pyloriscreening: Options and challenges. Expert Rev. Gastroenterol. Hepatol..

[41] Kaufman D.W., Kelly J.P., Wiholm B.-E., Laszlo A., Sheehan J.E., Koff R.S., Shapiro S. (1999). The Risk of Acute Major Upper Gastrointestinal Bleeding Among Users of Aspirin and Ibuprofen at Various Levels of Alcohol Consumption. Am. J. Gastroenterol..

[42] Li T.-K. (2008). Quantifying the risk for alcohol-use and alcohol-attributable health disorders: Present findings and future research needs. J. Gastroenterol. Hepatol..

[43] Cavendish R.L., Santos J.D.S., Neto R.B., Paixão A.O., Oliveira J.V., Araújo E.D., E Silva A.A.B., Thomazzi S.M., Cardoso J.C., Gomes M.Z. (2015). Antinociceptive and anti-inflammatory effects of Brazilian red propolis extract and formononetin in rodents. J. Ethnopharmacol..

[44] Souto E., Souto S.B., Zielińska A., Durazzo A., Lucarini M., Santini A., Horbańczuk O.K., Atanasov A., Marques C., Andrade L. (2020). Perillaldehyde 1,2-epoxide Loaded SLN-Tailored mAb: Production, Physicochemical Characterization and In Vitro Cytotoxicity Profile in MCF-7 Cell Lines. Pharmaceutics.

[45] Souto E., Zielińska A., Souto S.B., Durazzo A., Lucarini M., Santini A., Silva A.M., Atanasov A., Marques C., Andrade L. (2020). (+)-Limonene 1,2-Epoxide-Loaded SLNs: Evaluation of Drug Release, Antioxidant Activity, and Cytotoxicity in an HaCaT Cell Line. Int. J. Mol. Sci..

[46] Robert A., Nezamis J.E., Lancaster C., Hanchar A.J. (1979). Cytoprotection by prostaglandins in rats. Gastroenterology.

[47] Pinto L.A., Cordeiro K.W., Carrasco V., Carollo C.A., Cardoso C.A.L., Argadoña E.J.S., Freitas K.D.C. (2014). Antiulcerogenic activity of Carica papaya seed in rats. Naunyn Schmiedeberg’s Arch. Pharmacol..

[48] Djahanguiri B. (1969). The production of acute gastric ulceration by indomethacin in the rat. Scand. J. Gastroenterol.

[49] Gamberini M.T., Skorupa L.A., Souccar C., Lapa A.J. (1991). Inhibition of gastric secretion by a water extract from Baccharis triptera. Mem. Inst. Oswaldo Cruz.

[50] Shay H. (1945). A simple method for the uniform production of gastric ulceration in the rat. Gastroenterology.

[51] Sun X.-B., Matsumoto T., Yamada H. (1991). Effects of a Polysaccharide Fraction from the Roots of Bupleurum falcatum L. on Experimental Gastric Ulcer Models in Rats and Mice. J. Pharm. Pharmacol..

[52] Okunji C., Okeke C.N., Gugnani H.C., Iwu M.M. (1990). An Antifungal Spirostanol Saponin from Fruit Pulp of Dracaena mannii. Int. J. Crude Drug Res..

[53] Lobo V., Patil A., Phatak A., Chandra N. (2010). Free radicals, antioxidants and functional foods: Impact on human health. Pharmacogn. Rev..

[54] Silva A.M., Martins-Gomes C., Fangueiro J.F., Andreani T., Souto E.B. (2019). Comparison of antiproliferative effect of epigallocatechin gallate when loaded into cationic solid lipid nanoparticles against different cell lines. Pharm. Dev. Technol..

[55] Bhattacharyya A., Chattopadhyay R., Mitra S., Crowe S.E. (2014). Oxidative stress: An essential factor in the pathogenesis of gastrointestinal mucosal diseases. Physiol. Rev..

[56] Salatino A. (2018). Brazilian Red Propolis: Legitimate Name of the Plant Resin Source. MOJ Food Process. Technol..

[57] Raheja S., Girdhar A., Lather V., Pandita D. (2018). Biochanin A: A phytoestrogen with therapeutic potential. Trends Food Sci. Technol..

[58] Da Silva R.O., Andrade V.M., Rêgo E.S.B., Dória G.A.A., Lima B.D.S., Da Silva F.A., Araújo A.A.D.S., Júnior R.L.C.D.A., Cardoso J.C., Gomes M.Z. (2015). Acute and sub-acute oral toxicity of Brazilian red propolis in rats. J. Ethnopharmacol..

[59] Rocha N.F.M., De Oliveira G.V., De Araújo F.Y.R., Rios E.R.V., Carvalho A.M.R., De Vasconcelos S.M.M., Macedo D., Soares P.M.G., De Sousa D.P., De Sousa F.C.F. (2011). (−)-α-Bisabolol-induced gastroprotection is associated with reduction in lipid peroxidation, superoxide dismutase activity and neutrophil migration. Eur. J. Pharm. Sci..

[60] Lucarini M., Durazzo A., Kiefer J., Santini A., Lombardi-Boccia G., Souto E.B., Romani A., Lampe A., Nicoli S.F., Gabrielli P. (2019). Grape Seeds: Chromatographic Profile of Fatty Acids and Phenolic Compounds and Qualitative Analysis by FTIR-ATR Spectroscopy. Foods.

[61] Ribeiro A.R.S., Diniz P.B., Estevam C.S., Pinheiro M.S., Albuquerque-Júnior R.L., Thomazzi S.M. (2013). Gastroprotective activity of the ethanol extract from the inner bark of Caesalpinia pyramidalis in rats. J. Ethnopharmacol..

[62] Salehi B., Venditti A., Sharifi-Rad J., Kregiel D., Sharifi-Rad J., Durazzo A., Lucarini M., Santini A., Souto E.B., Novellino E. (2019). The Therapeutic Potential of Apigenin. Int. J. Mol. Sci..

[63] Sánchez-Mendoza M.E., Rodríguez-Silverio J., Rivero-Cruz J.F., Rocha-González H.I., Pineda-Farías J.B., Arrieta J. (2013). Antinociceptive effect and gastroprotective mechanisms of 3,5-diprenyl-4-hydroxyacetophenone from Ageratina pichinchensis. Fitoterapia.

[64] Hotta S., Uchiyama S., Ichihara K. (2020). Brazilian red propolis extract enhances expression of antioxidant enzyme genes in vitro and in vivo. Biosci. Biotechnol. Biochem..

[65] Osés S., Marcos P., Azofra P., De Pablo A., Fernández-Muíño M.Á., Sancho M.T. (2020). Phenolic Profile, Antioxidant Capacities and Enzymatic Inhibitory Activities of Propolis from Different Geographical Areas: Needs for Analytical Harmonization. Antioxidants.

[66] Rivero-Cruz J.F., Granados-Pineda J., Pedraza-Chaverri J., Rojas J.M.P., Passari A.K., Díaz-Ruiz G., Rivero-Cruz B.E. (2020). Phytochemical Constituents, Antioxidant, Cytotoxic, and Antimicrobial Activities of the Ethanolic Extract of Mexican Brown Propolis. Antioxidants.

[67] Svečnjak L., Marijanović Z., Okińczyc P., Kuś P.M., Jerković I. (2020). Mediterranean Propolis from the Adriatic Sea Islands as a Source of Natural Antioxidants: Comprehensive Chemical Biodiversity Determined by GC-MS, FTIR-ATR, UHPLC-DAD-QqTOF-MS, DPPH and FRAP Assay. Antioxidants.

[68] Govindasami S., Uddandrao V.V.S., Raveendran N., Sasikumar V. (2020). Therapeutic Potential of Biochanin-A Against Isoproterenol-Induced Myocardial Infarction in Rats. Cardiovasc. Hematol. Agents Med. Chem..

[69] Xue Z., Zhang Q., Yu W., Wen H., Hou X., Li D., Kou X. (2017). Potential Lipid-Lowering Mechanisms of Biochanin A. J. Agric. Food Chem..

[70] Jia W.C., Liu G., Zhang C.D., Zhang S.P. (2014). Formononetin attenuates hydrogen peroxide (H_2_O_2_)-induced apoptosis and NF-κB activation in RGC-5 cells. Eur. Rev. Med. Pharm. Sci..

[71] Fukai T., Marumo A., Kaitou K., Kanda T., Terada S., Nomura T. (2002). Anti-Helicobacter pylori flavonoids from licorice extract. Life Sci..

[72] Satoh H., Saijo Y., Yoshioka E., Tsutsui H. (2010). Helicobacter Pylori Infection is a Significant Risk for Modified Lipid Profile in Japanese Male Subjects. J. Atheroscler. Thromb..

[73] Gunji T., Matsuhashi N., Sato H., Fujibayashi K., Okumura M., Sasabe N., Urabe A. (2008). Helicobacter PyloriInfection Is Significantly Associated with Metabolic Syndrome in the Japanese Population. Am. J. Gastroenterol..

[74] Franchin M., Da Cunha M.G., Denny C., Napimoga M.H., Cunha F.Q., Bueno-Silva B., De Alencar S.M., Ikegaki M., Rosalen P.L. (2013). Bioactive Fraction of Geopropolis fromMelipona scutellarisDecreases Neutrophils Migration in the Inflammatory Process: Involvement of Nitric Oxide Pathway. Evid. Based Complement. Altern. Med..

[75] Kalil M.A., Santos L.M., Barral T.D., Rodrigues D.M., Pereira N.P., Sá M.D.C.A., Umsza-Guez M.A., Machado B.A.S., Meyer R., Portela R.D. (2019). Brazilian Green Propolis as a Therapeutic Agent for the Post-surgical Treatment of Caseous Lymphadenitis in Sheep. Front. Vet. Sci..

[76] Pineros A., De Lima M., Rodrigues T.S., Gembre A.F., Bertolini T.B., Fonseca M.D., Berretta A.A., Ramalho L.N.Z., Cunha F.Q., Hori J.I. (2020). Green propolis increases myeloid suppressor cells and CD4+Foxp3+ cells and reduces Th2 inflammation in the lungs after allergen exposure. J. Ethnopharmacol..

[77] Shedeed H.A., Farrag B., Elwakeel E.A., El-Hamid I.S.A., El-Rayes M.A.H. (2019). Propolis supplementation improved productivity, oxidative status, and immune response of Barki ewes and lambs. Vet. World.

[78] Usman A.N., Abdullah A.Z., Raya I., Budiaman B., Bukhari A. (2020). Glucocorticoid and cortisol hormone in response to honey and honey propolis supplementation in mild stress women. Enferm. Clín..

[79] Chen H.-Q., Jin Z., Li G.-H. (2007). Biochanin A protects dopaminergic neurons against lipopolysaccharide-induced damage through inhibition of microglia activation and proinflammatory factors generation. Neurosci. Lett..

[80] Ma Z., Ji W., Fu Q., Ma S. (2013). Formononetin Inhibited the Inflammation of LPS-Induced Acute Lung Injury in Mice Associated with Induction of PPAR Gamma Expression. Inflammation.

[81] Konturek P.C., Konturek S.J., Cześnikiewicz M., Płonka M., Bielański W. (2002). Interaction of Helicobacter pylori (Hp) and nonsteroidal anti-inflammatory drugs (NSAID) on gastric mucosa and risk of ulcerations. Med. Sci. Monit..

[82] Wallace J.L., McKnight W., Reuter B.K., Vergnolle N. (2000). NSAID-induced gastric damage in rats: Requirement for inhibition of both cyclooxygenase 1 and 2. Gastroenterology.

[83] Ribeiro D., Freitas M., Tomé S.M., Silva A.M.S., Laufer S., Lima J.L.F.C., Fernandes E. (2014). Flavonoids Inhibit COX-1 and COX-2 Enzymes and Cytokine/Chemokine Production in Human Whole Blood. Inflammation.

[84] Yu J., Bi X., Yu B., Chen D. (2016). Isoflavones: Anti-Inflammatory Benefit and Possible Caveats. Nutrients.

[85] Gracioso J.D.S., Vilegas W., Lima C.A.H., Brito A.R.M.S. (2002). Effects of tea from Turnera ulmifolia L. on mouse gastric mucosa support the Turneraceae as a new source of antiulcerogenic drugs. Boil. Pharm. Bull..

[86] De Barros M., Da Silva L.M., Boeing T., Somensi L.B., Cury B.J., Burci L.D.M., Santin J.R., De Andrade S.F., Monache F.D., Cechinel-Filho V. (2016). Pharmacological reports about gastroprotective effects of methanolic extract from leaves of Solidago chilensis (Brazilian arnica) and its components quercitrin and afzelin in rodents. Naunyn Schmiedeberg’s Arch. Pharmacol..

[87] Hou W., Schubert M.L. (2006). Gastric secretion. Curr. Opin. Gastroenterol..

[88] Xu M.L., Bi C.W., Kong A.Y., Dong T.T., Wong Y.H., Tsim K.W. (2016). Flavonoids induce the expression of acetylcholinesterase in cultured osteoblasts. Chem. Interact..

[89] Borrelli F., Izzo A.A. (2000). The plant kingdom as a source of anti-ulcer remedies. Phytother. Res..

[90] Atherton J.C. (2006). The Pathogenesis Ofhelicobacter Pylori–Induced Gastro–Duodenal Diseases. Annu. Rev. Pathol. Mech. Dis..

[91] Boyanova L., Derejian S., Koumanova R., Katsarov N., Gergova G., Mitov I., Nikolov R., Krastev Z. (2003). Inhibition of Helicobacter pylori growth in vitro by Bulgarian propolis: Preliminary report. J. Med. Microbiol..

[92] Baltas N., Karaoglu S.A., Tarakci C., Kolayli S. (2016). Effect of propolis in gastric disorders: Inhibition studies on the growth of Helicobacter pylori and production of its urease. J. Enzym. Inhib. Med. Chem..

